# A feasibility study of implementing grip strength measurement into routine hospital practice (GRImP): study protocol

**DOI:** 10.1186/s40814-016-0067-x

**Published:** 2016-06-06

**Authors:** Kinda Ibrahim, Carl May, Harnish P. Patel, Mark Baxter, Avan A. Sayer, Helen Roberts

**Affiliations:** 1 Academic Geriatric Medicine, Mailpoint 807, Southampton General Hospital, Tremona Road, Southampton, SO16 6YD UK; 2 NIHR CLAHRC: Wessex, Faculty of Health Sciences, University of Southampton, Highfield, Southampton, SO17 1BJ UK; 3 Medicine for Older People, Mailpoint 63, Southampton General Hospital, Tremona Road, Southampton, SO16 6YD UK; 4 Institute of Ageing and Institute of Health and Society, Campus for Ageing and Vitality, Newcastle University, Newcastle, NE4 5PL UK

**Keywords:** Older, Inpatients, Grip strength, Implementation, Clinical practice, Hospital

## Abstract

**Background:**

Handgrip strength is a non-invasive marker of muscle strength, and low grip strength in hospital inpatients is associated with poor healthcare outcomes including longer length of stay, increased functional limitations, and mortality. Measuring grip strength is simple and inexpensive. However, grip strength measurement is not routinely used in clinical practice. The aim of this study is to evaluate the feasibility of implementing grip strength measurement into routine clinical practice.

**Methods/design:**

This feasibility study is a mixed methods design combining qualitative, quantitative, and economic elements and is based on the acute medical wards for older people in one hospital. The study consists of three phases: phase 1 will define current baseline practice for the identification of inpatients at high risk of poor healthcare outcomes, their nutrition, and mobility care through interviews and focus groups with staff as well as a review of patients’ clinical records. Phase 2 will focus on the feasibility of developing and implementing a training programme using Normalisation Process Theory to enable nursing and medical staff to measure and interpret grip strength values. Following the training, grip strength will be measured routinely for older patients as part of admission procedures with the use of a care plan for those with low grip strength. Finally, phase 3 will evaluate the acceptability of grip strength measurement, its adoption, coverage, and basic costs using interviews and focus groups with staff and patients, and re-examination of clinical records.

**Discussion:**

The results of this study will inform the translation of grip strength measurement from a research tool into clinical practice to improve the identification of older inpatients at risk of poor healthcare outcomes.

**Trial registration:**

Clinicaltrials.gov NCTO2447445

**Electronic supplementary material:**

The online version of this article (doi:10.1186/s40814-016-0067-x) contains supplementary material, which is available to authorized users.

## Background

Older people are disproportionately represented among hospital inpatients. Nearly two thirds (65 %) of people admitted to a hospital in the UK are aged over 65 years old [1], occupying more than 51,000 acute care beds at any one time [2]. Frailty and multi-morbidity are very common among older hospital patients [3, 4]. Grip strength has been proposed as a useful single marker of physical frailty and biological ageing [5]. Lower grip strength is associated with higher hospitalisation costs and longer hospital stays among older people across the spectrum of clinical settings [6–11].

Grip strength is a key component of the diagnosis of sarcopenia, a common progressive and generalised loss of skeletal muscle mass and strength with a risk of adverse outcomes such as physical disability, poor quality of life, and death [12]. Sarcopenia is highly prevalent (25 %) among hospitalised inpatients [13]. Low grip strength (reduced muscle strength) is associated with poor current and future health including increased falls [14], increased risk of osteoporosis and fracture [15], coronary heart disease, and stroke [16], increased all-cause mortality [17], and reduced health-related quality of life in older people [18]. Grip strength cutoff points to identify those at risk have been proposed. For example, the European Working Group on Sarcopenia in Older People (EWGSOP) originally defined the cutoff points for identifying older people with sarcopenia to be < 30 kg for men and < 20 kg for women [19]. The Foundation for the National Institutes of Health (FNIH) Sarcopenia project reported that grip strength cutoff points which were associated with functional weakness were lower at < 26 kg for men and < 16 kg for women [20]. Recently, a meta-analysis of data from 12 UK cohorts has defined low grip strength as at least 2.5 SDs below the gender-specific peak mean [21]. From this study, low grip strength for people over 80 years old is defined as less than 27 kg for men and less than 16 kg for women, and these cutoff points will be used in our study.

Grip strength can be improved through physical exercise and nutritional supplements [22]. A Cochrane Review showed that progressive resistance training in particular could improve the strength, physical performance, and physical abilities of older community-dwelling people with no reported harmful side-effects [23]. Resistance training in older people with moderate dementia has also proved to be feasible leading to an increase in grip strength within 6 weeks [24]. Moreover, a 10-week programme of three times weekly progressive resistance training to regain muscle strength has been reported to be safe and effective among frail hospitalised older patients [25]. Nutritional interventions may also be beneficial to older adults with low grip strength. The prevention and treatment of sarcopenia require a sufficient amino acid and caloric supply [22]. A Cochrane Review has reported that grip strength was greater in those who received dietary advice and oral nutritional supplements compared with those who received dietary advice alone [26]. The PROT-AGE study group recommended average daily protein intake in older people of at least 1.0 to 1.2 g/kg body weight/day to maintain muscle mass and strength [27]. Older people with acute or chronic diseases require a higher dietary protein intake (i.e. 1.2–1.5 g/kg body weight/day).

The routine measurement of grip strength in older patients admitted to hospital could identify those who are at higher risk of functional decline and/or long length of stay and would enable appropriate interventions such as nutritional protein supplementation and resistance exercises to be implemented. There is limited evidence that trained clinical staff including nurses and physiotherapist can measure patients’ grip strength [28, 29]. Thus, the aim of this study is to assess the feasibility of implementing grip strength measurement and its relevant care plan into routine clinical practice and to identify factors that promote or inhibit this process.

### Study objectives

The specific objectives of this study are the following:

Objective 1: Define baseline practice in acute medical wards for older people in one hospital in relation to the identification of older inpatients at risk of poor healthcare outcomes and their current nutritional and mobility care.

Objective 2: Develop and deliver an educational training programme on grip strength measurement to clinical staff.

Objective 3: Monitor and evaluate routine implementation of grip strength by assessing the acceptability, adoption and coverage of routine GS measurement, and the basic costs of implementation.

Objective 4: Identify facilitators and barriers to the implementation process.

## Methods/design

### Study design

This feasibility study is a mixed methods design combining qualitative, quantitative, and economic elements. Our approach to intervention design and implementation evaluation will be informed by Normalisation Process Theory (NPT), focusing primarily on practice change, which addresses explicitly the issue of how interventions are adopted, embedded, and integrated into organisational routines [30]. NPT explains how interventions become routinely embedded in a context by referencing to four mechanisms: coherence or sense-making, cognitive participation, collective action, and reflexive monitoring. We will focus on practice change and how grip strength measurement is adopted and integrated into clinical routines [31]. This protocol complies with the SPIRIT guideline of writing protocols (see Additional file 1, SPIRIT 2013 Checklist).

The study will be conducted in five acute medical wards for older people in one hospital in England with 120 beds in total. All five wards admit unselected emergency medical patients aged 80 years and over and include two female wards, two male wards, and one mixed sex ward. The study comprises three phases with an explanatory sequential design, whereby the qualitative data will be used to gain better understanding of the quantitative findings (see the study flow chart in Additional file 2). In order to understand the embedding of a new practice into daily activities and to enhance *coherence* of the new practice, we will evaluate what people actually do and how they work. Thus, the first phase of the study will define the current practice on the wards with regard to how patients at high risk of poor healthcare outcomes are identified and their nutritional care and management of their mobility. Implementation research often focuses on the strategies necessary to deliver or implement interventions [32]. The intervention adopted in this research is “education and training”. Therefore, the second phase of the study involves developing and delivering an educational training programme on grip strength measurement to clinical staff. The findings from phase 1 and the NPT components will inform the development of the training programme. The training will be designed to enable clinical staff to make sense of the new practice (implementing grip strength measurement routinely), promote their understanding of the importance of measuring grip strength, and encourage their engagement in planning and delivering the implementation of grip strength measurement. The *Collective action* component of NPT defines and organises the enacting of a practice. This involves a review to ensure that the tasks are performed as required and that the work is allocated appropriately. In addition, any staff concerns about the work required will be acknowledged and they will be encouraged to share their ideas about how these might be managed during the training and later throughout the implementation process. The routine implementation of grip strength measurement will commence soon after completing the training. Participants’ *reflexive monitoring*, which defines and organises assessment of the outcomes of a practice, will be evaluated during the implementation process. The study period is summarised in Table 1.

**Table 1:** The study period according to SPIRIT figure

Description	Study period			
	T0 (3 months)	T1 (3 months)	T2 (3 months)	T3 (3 months)
Phase 1: Define baseline practice (interviews/ focus groups).	✓			
Phase 1: Define baseline practice (audit of patients’ medical records).	✓			
Phase 2: Design the training program and finalise the care plan for low grip strength.		✓		
Phase 2: Train staff on measuring grip strength and on the use of the care plan.		✓		
Phase 2: Routine implementation of grip strength measurement.			✓	✓
Phase 3: Assess outcomes of routine grip strength implementation (coverage).			✓	✓
Phase 3: Assess outcomes of routine grip strength implementation (Patients’ acceptability).				✓
Phase 3: Assess outcomes of routine grip strength implementation (staff acceptability and adoption).			✓	✓
Data analysis and dissemination.	✓	✓	✓	✓

### Phase 1: Baseline practice (meeting objective 1)

The aim of this phase is to define current baseline practice in acute medical wards with regard to the identification of patients at high risk of poor healthcare outcomes and their nutritional care and mobility. For this purpose, an ethnographic approach involving interviews, focus groups, and audit of clinical records will be followed. The central aim of ethnography is to provide rich, holistic insights into people’s views and actions, as well as the nature of the location they inhabit [33]. Understanding how the healthcare system works will enable the integration of grip strength measurement into routine practice in an effective way.

#### Semi-structured interviews or focus groups

Qualitative research studies usually involve a small sample size compared to quantitative research, but the data generated are substantial and detailed [34]. Some researchers suggest that the sample size should be around 20 to 30 [35] or the sampling should continue until reaching saturation level when no new concepts emerge from data analysis [36]. Following purposeful sampling, there are often some pre-determined criteria relating to sampling and participants are chosen based on the fulfilment of these standards [36]. We will conduct in-depth semi-structured interviews/focus groups with healthcare staff who are involved in the care of older people working in the five study wards. Staff participants will include those with different levels of experiences and roles including consultants, junior doctors, ward sisters, dieticians, physiotherapists, and therapy assistants. We aim to gain the individual views of 20–30 healthcare staff but will collect data until no new concepts are emerging.

Open-ended questions will be used to collect data on the current practice of staff with older inpatients, and written informed consent will be obtained. Three semi-structured interview/focus group schedules were developed to elicit information from relevant professional groups (medical and nursing staff, therapy staff, and dietetic staff) (Additional file 3). In general, questions will elicit (1) information on the current positions and professional backgrounds of each participant and their main roles, (2) how older inpatients are assessed on admission to the ward, (3) how patients at risk of poor healthcare outcomes are identified, and (4) what patient circumstances trigger dietetic or therapy input. At the end of the conversation, clinical participants will be introduced to grip strength measurement and we will obtain their initial views about using grip strength in clinical practice and identify any perceived potential facilitators and barriers for routine implementation of grip strength measurement. Focus groups will be preferred to gather shared information about the care provided to older patients, e.g. with nursing staff. However, individual interviews will be suitable to illicit the individual experience and practice, e.g. of medical consultants. Individual interviews will be used with participants who prefer private discussions or those who could not attend focus groups. The interviews/focus groups are anticipated to last less than 1 h and will take place in a private room in the hospital.

#### Audit of clinical records

Audit of a sample of clinical records will provide further evidence on whether the identification of patients at risk of poor healthcare outcomes occurs explicitly, which is documented and acted upon. Across the five study wards, a random sample of the clinical notes of 60 patients who would be eligible for grip strength measurement will be audited. All patients are considered eligible unless those who are in their terminal phase of illness or patients who have been at hospital for less than 3 days at the time of data collection. Basic information about each patient such as age, gender, date of admission, domicile status, and reasons for admission will be obtained. Information recorded within the first 3 days of admission about likely risk factors for poor healthcare outcomes including risk assessment measures applied to patients will be collected. These will include dietetic and therapy input, care plans in use, mobility level, history of falls, Malnutrition Universal Screening Tool (MUST) score, Do Not Resuscitate order (DNR), pressure ulcers assessment, recognition of dementia or delirium, and any other additional relevant information. Identifiable patient information will not be collected.

The number of referrals each week to the dietetic team from the study wards for the 3 months prior to the start of the study as well as the number of prescribed oral nutritional supplements (ONS) will be obtained from the hospital E-referral system and the hospital electronic prescribing system, respectively. This data will allow comparison to assess changes in routine practice following the implementation of the care plan for grip strength measurement. Patients are normally referred verbally and informally to the physiotherapy team; thus, obtaining the number of referrals to physiotherapy from the study wards for similar comparison is not currently possible.

### Phase 2: Training and implementation (meeting objective 2)

#### Develop and deliver a training programme

A training programme will be developed to provide nursing, medical, and therapy staff with the necessary knowledge and skills to implement grip strength measurement. Baseline clinical practice defined in phase 1 and the published literature about grip strength measurement will inform the training needs and the content of the training programme. Date from phase 1 will help us identify how best to integrate grip strength measurement in routine practice, e.g., who could do the measurement, how and where information about grip strength measurement can be documented and stored, and on the design and development of the training programme. In collaboration with nursing staff, therapy, and dietetic teams, we have developed a care plan for patients with low grip strength (Additional file 4). The training programme will be developed to match the constructs of the NPT (see Table 2) and include the following:

**Table 2:** Grip strength training programme based on Normalisation Process Theory (NPT)

NPT constructs	Training components and topics	Method
Coherence/sense-making	Understand the relevance of implementing grip strength measurement routinely. What are the cutoff points for grip strength, what to do with low grip strength levels. Introduction to the care plan for patients with low grip strength and enhance understanding the relevance of using the care plan for patients with low grip strength values. Introduction to Jamar dynamometer + practically measuring grip strength according to the standard protocol. Present the paperwork that need to be completed in relation to grip strength measurement and use of care plan.	Presentation Practical demonstration + video on grip strength measurement Presentation
Cognitive participation	Competence in grip strength measurement using the standardised protocol. Discussion about how to initiate and adopt grip strength implementation, talk about staff’ initial concerns about implementing grip strength, engage staff in identifying the best way to start and implement grip strength.	Supervised practical session of measuring grip strength of a colleague Group discussion
Collective action	Reaching a consensus about how to start and maintain implementation.	Group discussion
Reflexive monitoring	Discussion about how to engage staff in reflexive monitoring of grip strength implementation via sharing experiences and providing continuous feedback.	Presentation + group discussion

1. A presentation about grip strength and the clinical relevance of low grip strength values
2. An introduction to the care plan for managing patients with low grip strength
3. A practical demonstration of grip strength measurement using a Jamar dynamometer according to a standardised protocol (see Appendix 1) [37]

As grip strength measurement will be part of nursing admission procedures, nursing staff (*n* ≈ 150) across the five study wards will be trained in grip strength measurement in groups of two to six participants. The training sessions will be run daily for 4 weeks in each of the five study wards. We anticipate that training sessions will each last 20–30 min. Additional training sessions will be scheduled in collaboration with ward managers to train staff who could not attend during this period and new staff. The training programme will also be incorporated in the induction days of new student nurses and healthcare assistants. Additional training sessions will be provided to junior doctors, consultants, and therapy staff, incorporated into regular educational sessions where possible. The time and date of the training sessions will be agreed with the department training team to minimise disruption to the daily tasks of the clinical staff. At the end of each session, participants will be asked to formally evaluate the training session and give feedback.

Nurses attending the training session will be asked to measure the grip strength of a colleague according to the standard protocol as an assessment of their competency to measure grip strength of patients. Additionally, ongoing ward observation will be carried out by the research team and support will be offered to ensure nurses remain competent. Competency will be assessed in relation to positioning the patient correctly, giving verbal instructions to the patient, taking four measurements (two in each hand), recording the grip strength values, and completing a care plan as needed. The number, grade, and ward base of nursing staff attending training sessions and passing their competency assessment will be recorded. Nursing staff in each training session will be given the opportunity to express their initial views and any concerns about the training and the use of grip strength measurement in the routine assessment of older inpatients. This will also inform the content of subsequent training sessions.

#### Routine implementation of grip strength measurement

Implementation of routine grip strength measurement will start soon after completing the training in each ward with the aim that grip strength will be measured in all patients within 3 days of admission to the study wards as part of the admission procedure. If grip strength cannot be measured, e.g. an inability to hold the dynamometer in either hand (e.g. pain and/or severe arthritis), or inability to understand the explanation given (e.g. severe dementia or delirium), the reasons should be documented in the patient’s clinical records. Failure to complete the measurement will be managed as if the patient had low grip strength on the care plan. Patients who are in their terminal phase of illness will be excluded from the study.

Grip strength will be measured using a Jamar dynamometer by asking the patient to squeeze the dynamometer handle with each hand twice alternately, starting with the right hand using a standardised protocol [37]. A brief break of approximately 1 min will be allowed between each measurement, and the maximum value will be recorded in kilogrammes (kg). We will use two measurements with each hand instead of three measurements since our previous research with inpatients suggests that the third attempt is tiring and is rarely the maximum value. Patients unable to sit on a chair will still be included in the study and their grip strength will be measured according to the protocol but while the patient is sitting up in bed. The grip strength dynamometer will be calibrated at the beginning and end of the study and regularly every 2 months during the study period. Any damaged or faulty dynamometer will be replaced.

Patients who have low maximum GS values (men < 27 kg and women < 16 kg) or those who are unable to perform the test will receive a care plan. The care plan will focus on (1) dietary supplementation with oral nutritional supplements and (2) review of mobility by a physiotherapist to consider progressive resistance exercises to increase muscle strength (Additional file 4). We do not expect that measuring the patient’s grip strength will impose any risk to inpatients. However, we will deal with any complications resulted from study procedures as adverse events, recording their details and reporting them promptly.

### Phase 3: Monitoring and evaluation of routine grip strength implementation (meeting objectives 3 and 4)

Qualitative and audit data will be collected concurrently, analysed in real time and will be fed back to the clinical staff to inform ongoing change efforts. Monitoring and evaluation of routine grip strength implementation will involve assessing its acceptability, adoption, coverage, and costs. A summary of the study implementation outcome variables is presented in Table 3.

**Table 3:** Outcome variables for routine implementation of grip strength based on [32]

Implementation outcome variables	Definition	Assessment methods	
		Qualitative methods Interviews/focus groups	Quantitative methods Clinical audit
Acceptability	The extent to which the service is agreeable	+	
Adoption	The intention to try the service	+	
Coverage	The degree to which those with the greatest need received the service		+
Appropriateness	The relevance of the service	+	
Costs	Total cost of service in context		+

#### Semi-structured interviews/focus groups

We will collect qualitative data to assess the acceptability of grip strength measurement to staff and patients and adoption of routine grip strength measurement and identify the facilitators and barriers of the routine use of grip strength in clinical practice. Purposive sampling will be used to select a range of participants who have experience of grip strength measurement including patients and staff members.

Patient interviews:

A purposive sample of 10–15 patients, to include men and women with high and low grip strength across the study wards, will be invited by a member of their clinical team to take part in a short interview to assess the acceptability of grip strength test. Interviews will be conducted within 2 days of grip strength measurement to maximise recall. Patients will receive an information sheet describing the study and will have at least 24 h to decide whether they wish to participate further. Patients who do not have the capacity to consent will not be asked to participate in interviews. Interviews with patients are anticipated to last for 15–20 min. Patients will be asked open-ended questions about their views and experience of grip strength measurement in a semi-structured interview (Additional file 5).

Staff interviews/focus groups:

We will allow at least 4 weeks for the implementation of grip strength measurement routinely in the study wards prior to conducting interviews/focus groups. This will help us understand the acceptability of its implementation and how it was adopted and integrated in everyday work. A purposive sample of 10–20 clinical staff across the five study wards, including nursing staff, therapy, and dietetic teams, is anticipated to give a deep understanding of the experience of grip strength measurement. The questions will use NPT constructs to gain a view on how it has been operationalised and actioned across the five study wards (Additional file 5). All potential participants will receive an information sheet describing the study, and they will have at least 24 h to decide whether they wish to participate in the study. Prior to any interview/focus group, explicit written consent will be obtained from each participant. The interviews/focus groups are anticipated to last less than 1 h and will take place in a private room in the hospital.

#### Audit of clinical records

Quantitative data will be collected over the period of routine implementation to estimate reach or coverage of the routine grip strength measurement. The patients’ clinical records on each ward will be audited at regular intervals (at least every other week) to collect data on (1) the number of patients who have their grip strength measured and the range of values obtained, (2) the number of patients with low grip strength values, and (3) the number of patients with low grip strength who have received a grip strength care plan (see Table 4). These results should reveal the rate of progress of adopting grip strength measurement in routine practice. Results will be shared frequently with the study wards to encourage subsequent uptake.

**Table 4:** A grid to report weekly ward coverage of routine implementation of grip strength measurement

Bays/beds	Grip strength assessed (yes or no)	Grip strength		Has a Care plan been acted upon?			
		Maximum level	Assessed within 3 days (yes or no)	Care plan completed (yes or no)	ONS Prescribed (yes or no)	Referral to physio (yes or no)	Grip strength magnets (yes or no)
Bay 1							
Bed 1							
Bed 2							
Bed 3							
Bed 4							
Bed 5							
Bed 6							
Bay 2							
Bed 1							
Bed 2							
Bed 3							
Bed 4							
Bed 5							
Bed 6							
Bay 3							
Bed 1							
Bed 2							
Bed 3							
Bed 4							
Bed 5							
Bed 6							
Bay 4							
Bed 1							
Bed 2							
Bed 3							
Bed 4							
Bed 5							
Bed 6							

Once routine GS measurement is embedded in clinical practice for at least three months, the number of weekly referrals to the dietetic team and ONS prescriptions for the preceding 3 months will be extracted from the E-referrals and the electronic prescribing system and will be compared to the numbers collected at baseline.

#### Costs of implementation

The cost analysis will include the implementation costs and National Health Service (NHS) resource utilisation. The implementation costs will include the cost of equipment, staff training, and note audits. Resource use information will include nutritional prescriptions, referrals to a dietician, length of stay, and discharge destination. The results will be presented as the cost per patient and cost per unit of 120 beds.

### Data management and analysis

#### Qualitative data

All interviews and focus groups will be audio-recorded on a digital voice recorder, transcribed verbatim, and then anonymised. Each recording and transcript as word documents will be password-protected. Data collected will be analysed using thematic analysis to identify themes following the six phases proposed by Braun and Clarke (2006) [38]. The phases are familiarisation with the data, coding, searching for themes, reviewing themes, defining and naming themes, and writing up. A descriptive coding scheme will be developed from transcripts and based on participants’ perceptions and experiences. Two types of coding will be used: “open coding” to locate themes followed by “focused coding” to determine which themes repeat often and which represent unusual concerns. Coding will proceed in an iterative way with detailed memos linking emergent themes. The perceptions and views of different stakeholder groups will be compared. The thematic analysis for qualitative data collected in phase 3 of the study will be more focused and based on the four constructs of NPT to identify facilitators and barriers for implementing grip strength measurement, but the researchers will remain sensitive to any new concepts not covered by NPT that could emerge. A software program for analysing qualitative data (e.g. NVivo 10) will be used to facilitate data analysis.

#### Quantitative data

All data will be double entered into a password-protected computer database and assigned a unique identification number. Quantitative analysis will involve mainly descriptive statistics using the statistical software package IBM SPSS statistics 22. Descriptive statistics will be used to report the data abstracted from the clinical records, the E-referral system, and the electronic prescribing system. Descriptive data will be summarised using mean and standard deviation (SD), median and inter-quartile range (IQR), and/or number (percent) as appropriate for the type of data (continuous, normally distributed or not, categorical).

The feasibility of training the clinical staff will be reported using descriptive statistics. This will include description of the trainees’ numbers, discipline, grade, team or ward base, degree of competence to measure grip strength, and their evaluation of the training received allowing comparisons to be made between trainees across the five study wards. Descriptive statistics will also be used to describe the coverage of grip strength implementation such as the number and proportion of patients who had their grip measured, had low grip strength values, received a care plan, the range of grip strength values, and the participants’ characteristics for each ward. This will allow comparisons to be made of the practice and implementation of grip strength measurement across the different wards. Chi-squared tests will be used to assess the coverage of routine grip strength assessment across the five wards, the number of referrals to the dietetic team, and the number of ONS prescription before and after the routine implementation of grip strength measurement.

### Overall mixed methods integration

The qualitative and quantitative results will be integrated; we will consider each analysis (qualitative and quantitative) on its own terms and how the two differ or converge in their findings when presenting the overall conclusion. Qualitative and quantitative data will be integrated through triangulation to examine (1) convergence (so results provide the same answer to the same questions), (2) expansion (are the findings collected from one data explained by another), and 3) complementary aspects (does embedding the results of one data within the other data set help contextualise overall results) [39]. The implementation costs (e.g., the training costs and costs of equipment) and NHS resource utilisation (e.g. ONS prescriptions) will be integrated in the final analysis and reports alongside the qualitative and quantitative data.

### Trial status

At the time of submission, data collection has started for phase 1. No data cleaning or analysis has been carried out.

## Discussion

The purpose of this study is to evaluate the feasibility and acceptability of implementing grip strength measurement into routine clinical practice to identify older patients at risk of poor healthcare outcomes. Many studies have demonstrated that older hospital patients with low grip strength have an increased risk of functional decline, long length of stay, admission to care homes, and death. Early identification of patients with low grip strength at admission to hospital will allow the possibility of appropriate early intervention. Translating this evidence-based research tool into clinical practice has the potential to improve the care of older patients. This mixed methods study will provide a rich picture of barriers and facilitators to the use of grip strength measurement routinely in an acute medical setting. The inclusion of purposive samples of clinicians and patients will capture the complexity of the roles and responsibilities that influence the implementation process and user views and experiences. If routine grip strength measurement is feasible and acceptable, this study will inform the implementation of grip strength assessment routinely in clinical practice in other hospitals and organisations.

## Appendix

### Standard protocol for measuring grip strength [32]

1. Sit the participant comfortably in the chair with their forearms on the arms of the chair and their wrist just over the end of the arm of the chair—wrist in a neutral position, thumb facing upwards. Feet flat on the floor.
2. Demonstrate how to use the dynamometer to show that gripping very tightly registers the best score.
3. Starting with the right hand position, the thumb around one side of the handle in position 2 and the four fingers are around the other side. The instrument should feel comfortable in the hand: alter the position of the handle if necessary.
4. Rest the base of the dynamometer on the palm of the observer’s hand as the participant holds the dynamometer. The aim of this is to support the weight of the dynamometer, but be careful not to restrict the “movement” of the machine.
5. Encourage the participant to squeeze as long and as tightly as possible or until the needle stops rising. Use a standard encouragement “and squeeze as tightly as you can”. Once the needle stops raising, you can instruct the participant to stop squeezing as they have achieved their peak.
6. The observer should read from the outside dial which gives grip strength in kilogrammes. Record the result to the nearest 1 kg on the data entry form.
7. Repeat measurement in the left hand.
8. Do one further measurements at least 1 min apart in each hand alternating sides to give two readings in total for each side.
9. For analysis, use the maximum grip score from each hand.
10. Clean the dynamometer with an alcohol wipe between patients and place the dynamometer back in its case.

## Additional files

Additional file 1: SPIRIT 2013 Checklist: Recommended items to address in a clinical trial protocol and related documents*. (DOC 121 kb)
Additional file 2: The study flow chart to illustrate the three study phases. (DOCX 25 kb)
Additional file 3: Semi-structure interview/focus group schedules for phase 1: define baseline practice. (DOCX 16 kb)
Additional file 4: Care plan for grip strength measurement. (PDF 350 kb)
Additional file 5: Semi-structured interview/focus group schedule for phase 3: monitoring and evaluation of routine implementation of grip strength measurement. (DOCX 18 kb)

## Abbreviations

NPT: Normalisation Process Theory
ONS: oral nutritional supplements
