# Supplementary material for: A feasibility study of implementing grip strength measurement into routine hospital practice (GRImP): study protocol
Source: Pilot Feasibility Stud. 2016 Jun 6;2:27. doi: 10.1186/s40814-016-0067-x (PMC5154137; doi:10.1186/s40814-016-0067-x)
Supplement: Additional file 2: — The study flow chart to illustrate the three study phases. (DOCX 25 kb) [file 40814_2016_67_MOESM2_ESM.docx]

**The Study flow chart to illustrate the three study phases**.

**Semi-structured Interviews/focus groups** with consultants, junior doctors, nursing staff, dieticians, physiotherapists, and therapy assistants (n=20-30).

**Review of patients’ clinical records** (60)

Identify number of and reasons for **referrals to dietetic/nutrition team** in the study wards for the last 3 months

Identify **the percentage of ONS prescribed** in the study wards for the last 3 months.

**Phase 1. Baseline practice**

*Identifying patients at high risk of poor outcomes

* The nutritional and mobility care provided to inpatients in acute medical wards for older people

**Semi-structured interviews/focus groups** with patients (n=10-15) and healthcare staff (n=10-20) to assess acceptability of grip strength measurement and adoption.

**Regular auditing of the clinical records (**the number of patients who have their grip strength measured, the number with low grip strength values, the number of patients who have received the grip strength care plan)

Identify **number of and reasons for** **referrals to dietetic/nutrition team** in the study wards for the last 3 months

Identify **the percentage of ONS prescribed** in the study wards for the last 3 months.

identify **implementation costs** including the cost of equipment, staff training, notes audit, ONS prescriptions and length of stay.

**Phase 3. Monitoring and evaluation of routine grip strength implementation**

Monitoring and evaluation of routine grip strength implementation will involve assessing its acceptability, adoption, coverage, and costs.

**Phase 2. Training and implementation**

* Develop and deliver training programme.

* Start routine implementation of grip strength measurement

**Develop a training programme + care plan** for managing patients with low grip strength

**Train the staff** in groups of 2-6 **(**presentation and practical demonstration) about measuring grip strength and use of the care plan

**Routine implementation of grip strength measurement**; measure grip strength for newly admitted patients in the study wards + use care plan for patients with low grip strength
